# Pharmacist-led clinical medication review service in primary care: the perspective of general practitioners

**DOI:** 10.1186/s12875-022-01963-w

**Published:** 2023-01-10

**Authors:** Urska Nabergoj Makovec, Tanja Tomsic, Mitja Kos, Tea Stegne Ignjatovic, Antonija Poplas Susic

**Affiliations:** 1 University of Ljubljana, Faculty of Pharmacy, Department of Social Pharmacy, Askerceva cesta 7, 1000 Ljubljana, Slovenia; 2 Community Health Centre Ljubljana, Ljubljana, Slovenia

**Keywords:** Clinical medication review, Clinical pharmacist, General practitioner, Primary care, Pharmacotherapy

## Abstract

**Background:**

An advanced level medication review service (CMR) is systematically reimbursed and available nationwide in Slovenian primary care since 2016. CMR is performed by clinical pharmacists (CP). Close collaboration with general practitioner (GP) is required as they perform patient selection and make clinical decisions regarding patient’s medication.

**Methods:**

A prospective observational study was conducted in 2018 aiming to evaluate the perspective of GPs on the implementation of pharmacist-led medication review service in Community Health Care Centre Ljubljana, Slovenia. GPs of the patients, who provided written informed consent were invited for the interviews. The semi-structured interview consisted of 5 open ended questions addressing reasons for referral of the patients, implementation of CP recommendations and the GPs’ perspective of the service in general. Interviews were audio recorded with GPs written consent, transcribed verbatim and inductive content analysis was performed in NVivo11 Pro.

**Results:**

In total 38 interviews with 24 GPs were performed. The emerged themes were nested under 3 main domains representing Donabedian model of quality healthcare – structure, process, outcomes. The service structure is built on broad pharmacotherapy knowledge as the main CP competency, good accessibility, and complementarity of healthcare professions. Patients are mainly referred to the CMR due to polypharmacotherapy, however in majority there is a more in-depth reason behind (e.g., adverse events, etc.). Lack of time to recognize eligible patients and additional workload to study and implement the recommendations present the major challenges in the service process and therefore low number of referrals. CPs recommendations are mostly accepted, although the implementation time varies. When recommendation addresses medicines prescribed by a clinical specialist, the CMR report is forwarded to them for decision regarding implementation. The empowerment of the patients in medicines use was emphasized as the major benefit of the CMR, which consequently supports and enhances the quality of GP’s patient care. Transferability of recommendations to similar cases and high satisfaction with the service of GPs and patients, were mentioned.

**Conclusion:**

GPs experiences with CMR are encouraging and supportive and present a base for further growth of the service.

**Supplementary Information:**

The online version contains supplementary material available at 10.1186/s12875-022-01963-w.

## Background

Clinical or advanced medication review (CMR) is the most comprehensive type of medication review (type 3 according to Pharmaceutical Care Network Europe (PCNE) typology), where appropriateness of patient’s medicines is evaluated based on medication history, review of medical records (clinical data) and conversation with the patient [1]. CMR is performed by (clinical) pharmacist (CP); however, physicians are the key stakeholders in selecting patients and clinical decision making [2–5]. Clear role specification, trustworthiness and professional interaction are the three elements that contribute to good collaborative care [3]. Useful and quality recommendations regarding patient’s medicines help build pharmacist expertise in physician’s eyes and enable professional interaction and communication [5].

CMR is implemented in several countries worldwide at different levels of healthcare [2, 6, 7]. In Slovenia the CMR is performed in hospitals as well as in primary care involving several community health centres and elderly residents’ homes. Following positive experiences and outcomes from the regional pilot project in 2012 [8–10], the service was implemented nationwide in 2016 with defined standard operating procedure [11, 12] and reimbursement [10]. The aim of the service is to support general practitioners (GPs) in rationalization and optimization of the individual patient medicines. GP refers the patient to a consultation with the CP. After consultation and medical records review, CP writes recommendations in a CMR report and sends it to a GP, who decides what to implement. In case, patients are unable to properly communicate the conversation can be performed with the patients’ carer or medication review is based only on information from the medical records (PCNE type 2b). Pharmacist, eligible to provide the service, must be licenced pharmacist, specialist of clinical pharmacy with gained extra competencies in a purposively designed educational programme or by establishing their experiences through an examination [11].

The nationwide implementation of CMR presented a change in the Slovenian primary healthcare and brought potential new aspects and stakeholders. When change is implemented, understanding the process and views of involved stakeholders is crucial to reach service full potential and sustainability [13–15]. Several studies, have assessed implementation of CMR in primary care from different perspectives, including the GPs [16–22]. In general, positive attitude and experiences were reported, although several barriers (lack of time, workload etc.) were also identified [16, 18, 20, 22, 23]. Majority of these evidence was gathered in healthcare systems where the pharmacist’s role in a healthcare team is strongly enrooted (e.g., UK, the Netherlands, Australia) [6]. Although, the main characteristics and objectives of the CMR are often similar [2, 6, 7], factors like the differences in healthcare system, perception of the pharmacist role in patient care, professional development background, reimbursement of the service might influence the implementation and sustainability of the service [3, 24, 25]. Therefore, we designed an observational study to gain an in depth understanding of GPs’ experiences with the CMR in the largest Slovenian community healthcare centre. The objective of the study was to describe the implementation of the CMR from the GP’s perspective by identifying facilitators, barriers and other implementation factors, influencing the provision of the CMR in Community Health Care Centre Ljubljana.

## Methods

### Study design and setting

A prospective observational study with qualitative approach was designed and conducted in collaboration between Community Health Centre Ljubljana (CHC LJ) and University of Ljubljana, Faculty of Pharmacy (UL FFA). Data collection was performed in CHC LJ, a public primary healthcare institution and largest community health centre in Slovenia. The centre employs around 150 GPs (regular and temporary) and takes care for 250.000 people within the Ljubljana municipality [26].

### Research team

The research team consisted of five members: (i) experienced pharmacists, specialist of clinical pharmacy, employed full-time as a CMR provider in the CHC LJ, (ii) practicing GP, specialist of family medicine in the CHC LJ and medical director of the CHC LJ, (iii) practicing GP (concessionary practice), specialist and professor of family medicine and head of Primary Healthcare Research and Development Institute (a unit within the CHC), (iv) professor of social pharmacy at UL FFA and senior researcher of pharmacy practice and pharmaceutical care, actively involved in the process of developing the concept of pharmaceutical cognitive services in Slovenia, (v) a PhD student at UL FFA researching implementation of cognitive pharmaceutical services in Slovenia. All members participated in the development of the interview guide, coordination of study-related activities and data analysis. The CP role was to invite and enrol patients and to coordinate with the PhD student (researcher), who was enrolling GPs and performing interviews. To preserve GP’s anonymity the research team from CHC LJ was not involved in the interview process and only accessed anonymized and aggregated data.

### Selection of participants

**Fig. 1:**
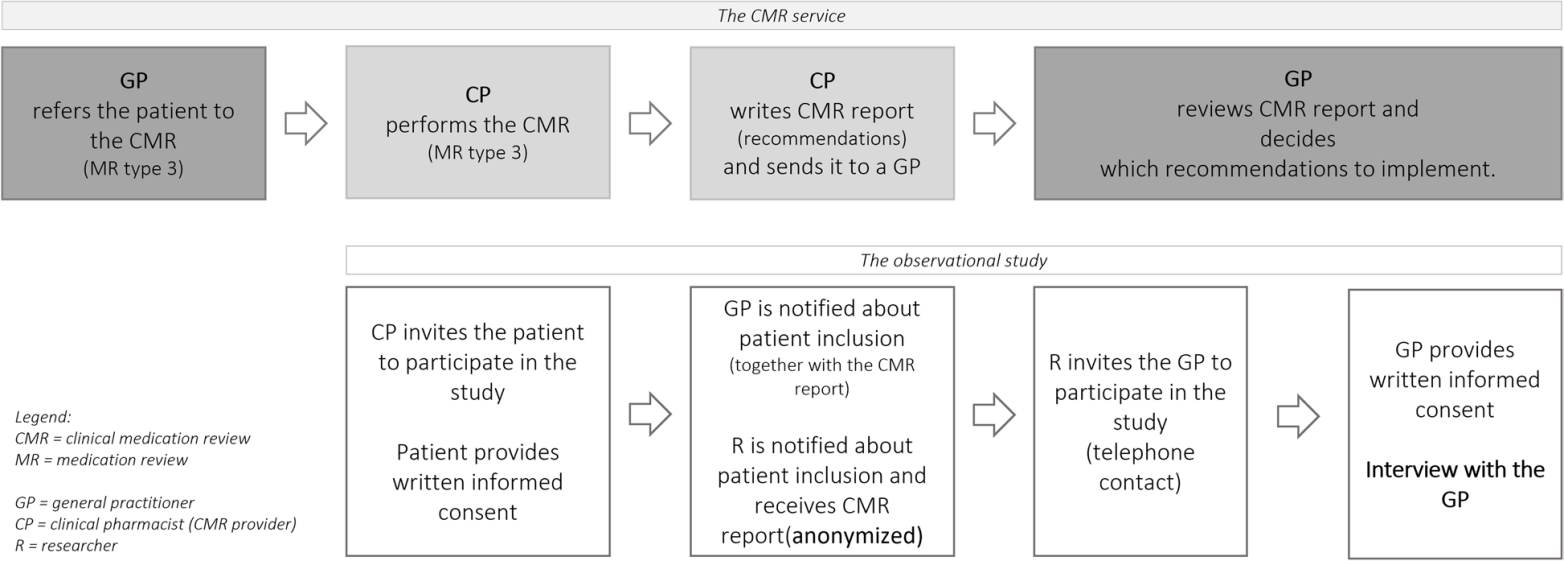
Presentation of the service (upper line) [11] and study (bottom line) process

A consecutive sampling technique was used. GPs were eligible to participate in the study if (i) they referred a patient to CMR service including conversation with the CP (type 3) (ii) patient provided written informed consent. Patients were excluded if (i) the conversation with the CP was performed only with their carer (ii) the patients were attendinga follow up visit with the CP at the time of enrolment (iii) patient’s cognitive or physical functions were severely impaired based on CP’s observation. At the beginning of the study, a circular was published, to notify all practicing GPs and other healthcare personal within CHC LJ about the study. A detailed study process is presented in Fig. 1*.* One GP could participate in several interviews if he/she referred more than one patient in the study period. At the first interview, GPs provided written informed consent and demographic data (gender, age, CHC LJ unit, number of patients, years of GP practice and number of patients referred to CMR).

### The interview guide

Prior to the study an interview guide was developed to comprise three major topics: (i) reasons for referral of the patients, (ii) assessment of the CMR report with implementation of CP recommendations and prediction of future treatment steps and (iii) the GPs’ perspective of the service in general. Five open-ended questions were formulated together with a set of sub-questions to be used if needed; the question are presented in the supplementary material (Additional file 1). The interview guide was based on research questions and experiences of the research team. No theories, frameworks or models were used in the development.

### The interviews

The interviews took place at the GP office, preferably within a week of the phone call, depending on GP’s availability. In case of multiple patients per GP in close time range, several interviews could be performed within one scheduled meeting. The interviews were semi-structured, adapted case by case using the interview guide. GPs’ perspective of the service in general was addressed only at the first interview in case of multiple interviews per GP. Each interview was audio recorded as a single recording; the researcher also took field “paper and pencil” notes during the conversation. The content of interviews and emerging ideas were frequently discussed within the research team during the study period.

### Qualitative analysis

Audio recordings were transcribed verbatim. Anonymized CMR reports and interview transcripts were imported in the QSR International NVivo 11 Pro software for inductive content analysis to systematically review, contextualize and condense participants responses (“raw data”) without applying frameworks or theories to a-priori formulate context [27]. In the present study, coding sentence by sentence was the first step to obtain meaningful themes (“free nodes”), which arose from similar responses by multiple participants. The next step was nesting, where similar themes were joined into sub-categories (“parent nodes”) and main categories (“tree nodes”) resulting in three levels of results. In discussion of the results, the elements of Donabedian model of healthcare [28] were recognized, which generated additional, fourth level (domains). Additionally, each CP recommendation and GP’s response to it were coded as cases and relationship between both (CP recommendation-GP response) was created to investigate the level of acceptance and implementation of the CP recommendations by the GPs. Nesting process was applied to create parent relationships depicting different layers of acceptance and implementation (accepted, partially or conditionally accepted, not accepted and the recommendation does not require an intervention or decision of the GP).

Firstly, the PhD student coded all the content, which was then reviewed and revised by the senior pharmacy researcher. Coding was then discussed, disagreements resolved, the nesting process agreed on and the idea to apply the Donabedian model was implemented. Following, the results were sent to the rest of the research team for reflection and finalization. The need to conduct member checking was not observed and therefore not performed [29].

### Presentation of results

Results were translated from Slovenian to English for presentation in the international literature. The translation was conducted by the research team, phrasing was adjusted to the type of language used during the interviews. To ensure adequate reporting of the study process and findings, consolidated criteria for reporting qualitative research (COREQ) were followed and are presented in the supplementary material (Additional file 2) [30].

## Results

### Sample size and response rate

During the study period (February to June 2018), 179 patients were referred to the CMR, 97 for review of medical documentation (type 2b) and 82 for conversation with the CP (type 3). CP invited 62 patients to participate and 40 provided written informed consent (65% response rate). Reasons for exclusion of the 20 patients are stated in the supplementary material (Additional file 3). The included patients were referred by 26 different GPs of whom 24 decided to participate (92% response rate) resulting in 38 performed interviews. Two GPs declined participation in the study due to objective reasons (office transfers and managing annual holiday substitutions). An average duration of an interview was 7.5 minutes; 17 GPs were interviewed once, 3 GPs were interviewed twice, and 4 GPs were interviewed 3–5 times.

### Characteristic of the participants

All six CHC LJ units were covered and distribution between them was even. On average participating GPs were responsible for care of 1900 patients (250–2500). Detailed characteristics are provided in Table 1.

**Table 1:** Description of the participating GPs (*N* = 24)

Characteristics	***N***	%
**Gender**		
*Female*	22	92
*Male*	2	8
**Age [years]**		
*below 40*	8	34
*41–50*	7	29
*51–60*	7	29
*over 60*	2	8
**Education**		
*Specialist of family medicine*	23	96
*Resident of family medicine*	1	4
**Working experience as GP [years]**		
*less than 5*	1	4
*5–10*	5	21
*10–20*	5	21
*20–30*	6	25
*more than 30*	7	29
**Position**		
*Primary GP (practice licence holder)*	20	83
*Substitution GP*	3	13
*Elderly resident home*	1	4
**Experience with CMR (N of referred patients)**		
*Less than 10*	10	42
*10–30*	11	46
*More than 30*	3	12

### Results of the qualitative content analysis

**Fig. 2:**
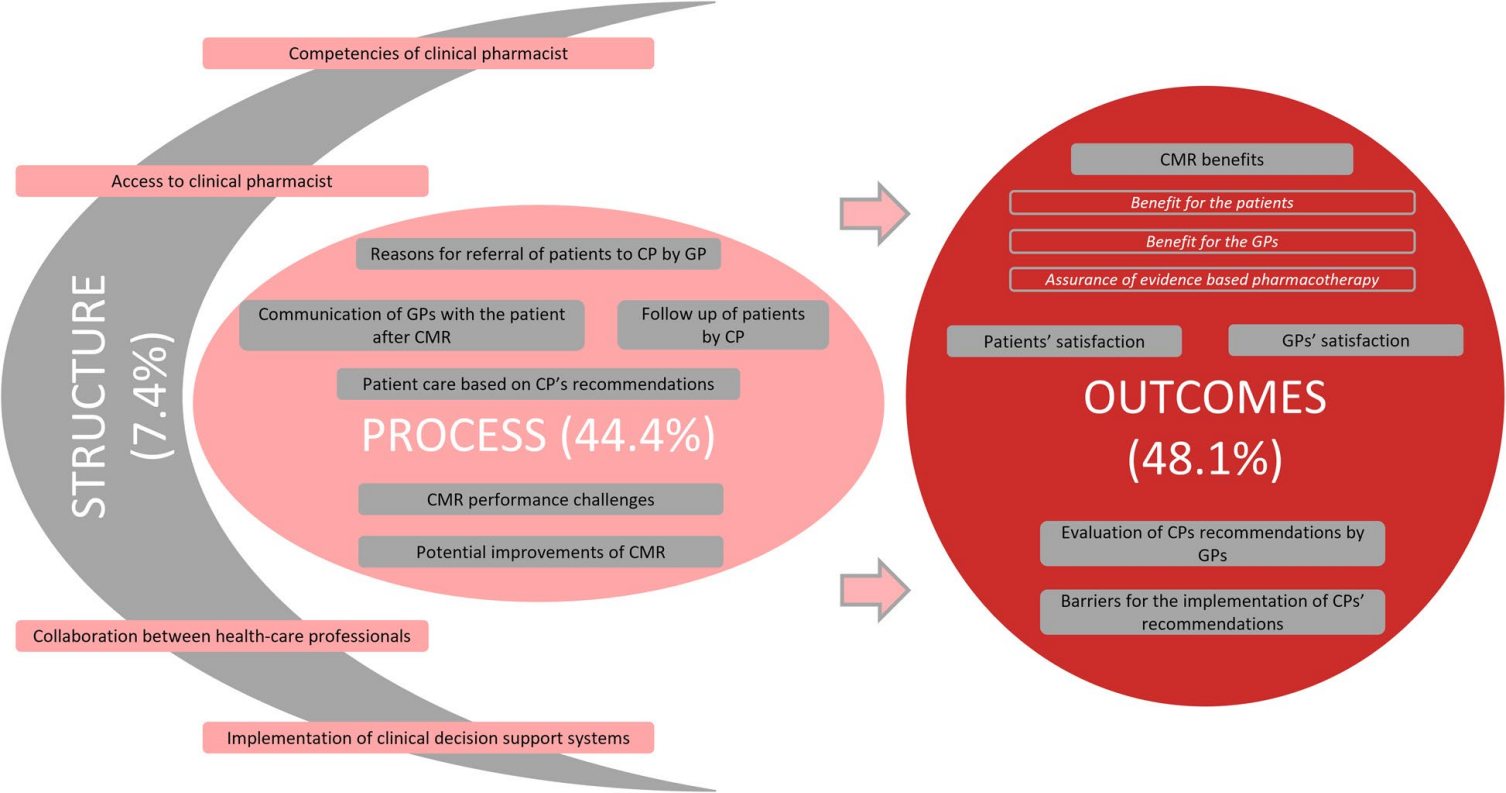
The visual representation of content analysis findings presented as the Donabedian quality of care [28]. The picture shows Donabedian quality of health care model [28] as a mind map, representing 3 main domains (structure, process, outcomes) of results. Percentages (%) next to the 3 domains show the proportion of different identified themes included in a domain. The oval filled shapes in the domains represent the main categories generated in the nesting process and nested under the same domain. The next level of results (sub-categories) are only indicated as an example in the unfilled oval shapes under outcomes and benefits of the CMR service

In total, 833 different themes emerged from the transcripts content analysis. In the nesting process 48 sub-categories were formed, which were further grouped in 15 main categories and finally in three domains representing the Donabedian quality of healthcare model - structure, process, outcomes (Fig. 2) [28].

#### Structure

Four categories represent the structure domain and are shown with citation examples in Table 2.

**Table 2:** Four categories included under the structure domain with GPs’ citations

Categories under structure domain	***Statement***	GP
**Competencies of clinical pharmacist**	*We are GPs and we practice family medicine. CP has its own role as he has much more knowledge and information about medicines pharmacokinetics, pharmacogenomics, interactions, adverse reactions, … We have some knowledge, but it is not that deep. It is a role that is irreplaceable in my opinion. We will always have the need for.*	GP 1
	*… we see different things and maybe don’t think the problems could be related to medicine. The pharmacist looks at the patients from a different perspective …*	GP 2
	*The main advantage is that patients tell them a lot of things, they do not tell me.*	GP 3
	*CP has more time for a more in-depth review.*	GP 4
**Access to clinical pharmacist**	*If I have any questions, I know CP is available also* via *email or* via *telephone for urgent matters …*	GP 5
	*It practical – in the same house, we know when she’s here.*	GP 6
**Collaboration between health-care professionals**	*We do not judge each other work, we work from two different perspectives, what is synergistic and positive for the patients.*	GP 1
	*It is an add on, to community pharmacists and to us. We all work together in satisfaction of the patients.*	GP 7
**Implementation of clinical decision support systems**	*There is something …*. *computer is throwing out something, but in my case it does not even work, I can read it and even if I could there is usually no time for that.*	GP 8

CP greatest competency is broad and up to date pharmacotherapy knowledge, which enables the CPto review patients’ medicines efficiently, from a different angle and provide fresh ideas for therapy optimization. Furthermore, the communication between CP and patient is more open, truthful and less time pressured. The access to CP is good. The CMR is recognized as an interprofessional collaboration and completion of two healthcare professions.

#### Process

Six categories represent the process domain and are shown with citation examples in Table 3.

**Table 3:** Six categories included under the process domain with GPs’ citations

Categories under process domain	***Statement***	GP
**Reasons for referral of patients to clinical pharmacist by general practitioner**	*The patient came to the nurse for prescription renewal and muttered about taking to many medicines. The nurse proposed the CMR.*	GP 3
	*The patient takes a lot of medicines, has kidney insufficiency and is sensitive to medicines changes. So, I wished that we lower the risk in prescribing as much as possible.*	GP 9
	*… patients with many Rx medications and adverse reactions. They present the biggest challenge.*	GP 10
	*… special patients’ groups like kidney insufficiency, liver insufficiency, pregnant women …. We don’t know much about new biological target medicines or patients on chemotherapy... Groups of patients with unusual therapy for a GP office …*	GP 9
	*I think the patient was referred to the CMR* via *anticoagulation office … It was not done by me …*	GP 11
**Communication of general practitioner with the patients after clinical medication review**	*Yes, we were in contact* via *telephone and email. The patient was not here in person though, which is of course different.*	GP 12
	*In this case we will need some more time to explain everything to the patient as she is very attached to her medicines. We didnot explain it by phone, we invited her to come in.*	GP 13
	*We have not yet scheduled the next visit and the things are also not of the serious nature …. So, in few months we will talk about it.*	GP 14
	*Sometime in May [CMR was performed mid-March], when medicines run out. We can’t even make appointments earlier …*	GP 10
**Patient care based on clinical pharmacist recommendations**	*The patient has the control visit already scheduled, so we will start with the implementation then.*	GP 15
	*We will check cholesterol … He won’t be taking statins for 6 weeks not, so we will see what the starting point will be. And then how much it will be decreased …*	GP 16
	*… if the values will raise three times above the upper reference limit …. We will send the patient to the diabetes clinic prematurely to change the medication …*	GP 3
**Follow up of patients by a clinical pharmacist**	*It depends how successful will I be with the implementation. If it will be ok, I don‘t see the need. If it doesn‘t work out, then probably.*	GP 10
**Clinical medication review service performance challenges**	*It looks like we [GPs] are not interested, however when you have 70 patients a day …*	GP 17
	*It’s all about time, to critically assess every patient in front of you and decide if you need to refer him. And then I guess there would be more referrals.*	GP 18
	*We will see … The patient has multiple medicines for this condition, I believe we could discontinue that one …. However, we need time to do that, and we don’t have it …*	GP 10
	*You know, there is a lot of work with this. If you want to refer someone, its additional workload …*	GP 19
	*I believe that when we get used to each other it will be better … we got used to it already [during the pilot project] and then there was the gap and it got out of routine ….*	GP 13
	*Sending medical documentation around is risky, I might need it in between, …*	GP 20
	*The CP was able to talk with this patient … But with many others it is not possible – dementia, decreased cognitive function … they are not able to communicate.*	GP 21
**Potential improvements of clinical medication review service**	*When I get the reports, I see how useful they are. And then more time you refer, more time you remember. Because you are satisfied with the feedback information and then you recall referring more times …*	GP 17
	*We had the idea for the CP to come during office hours to select patients … but currently the CP is not allowed to do that.*	GP 15
	*We need to be more aware that we have a CP in the house and be reminded more times.*	GP 9
	*… when they will see how good this is … every novelty is at first “phaa” … why would anybody stick around my patient records … but then ….*	GP 6
	*It would be nice if the CP could notify us about changes of medicines at the market … It is done once a month, at the meetings, but sometimes it would be useful to notify us right away by email so that we don’t send patients up and down.*	GP 13
	*To cite and reference more of clinical studies … like she did in this case …*	GP 14
	*It would be nice to personally talk with the CP sometimes …*	GP 22

Polypharmacotherapy was the most common stated reason on the referral form and it was also the actual reason when referral was based on patient’s wish to decrease number of medicines. The reasons were more specific (e.g., adverse reaction, interactions, poor kidney function …) when referrals were based on the GP’s initiative. Some GPs even established their own criteria based on which they refer patients. The first contact of GP with the patient after CMR happened soon after GP’s review of the CMR report, either in person or by phone. The nature and seriousness of the recommendations and GPs’ workload influenced how fast the first contact occurred. The implementation time of the accepted recommendations varied from 1 to 3 months, usually starting at the first scheduled regular visit. Future care encompassed laboratory monitoring, referrals to clinical specialist, while follow up visit with CP of the same patient were only reported if new DRPs or questions arose. Work overload and lack of time were identified as the two major barriers in CMR implementation and performance. GPs underlined that they need time to assess and properly select the patients as well as to study the CP recommendations and implement them. Therefore, CMR also represents additional workload and time for GP. Other identified barriers were CMR is not yet embedded in GP’s daily routine, non-digital patients’ medical records and patients’ cognitive function, which only enable provision of the type 2b CMR, especially in the elderly residential homes.

GPs recognized the number of referred patients is low and should be increased. Positive past experiences were mentioned as motivation and reminder to refer patients more often. Several ideas how to increase the number of referrals were provided - (i) more time for the conversation with the patients, (ii) criteria for automatic referrals (iii) authorization of CP or nurses for the selection of eligible patients, (iv) reminder systems, (v) higher activation of the GPs in elderly resident homes, (vi) raising awareness among GPs, who haven’t yet had experiences with the CMR. Other improvements (regular pharmacotherapy updates by the CP, changes of the form and content of the reports, better access to the CP) were also proposed.

#### Outcomes

Five main categories represent the outcomes domain and are shown with citation examples in Table 4.

**Table 4:** Five categories included under the outcome domain with GPs’ citations

Categories under outcomes domain	***Statement***	GP
**Evaluation of clinical pharmacists’ recommendations by general practitioners**	*This about lercanidipine was useful, that if we would titrate the dosing slower, the patient might have not had problems. Its good warning for the next time.*	GP 6
	*The advice was to try ezetimibe and I really haven’t tried it yet. So yes, I will try that.*	GP 16
	*It’s interesting … the CP suggested to decrease the number of medicines with introduction of three active substances in one pill … I found the advice very suitable.*	GP 3
	*The patient has diabetic polyneuropathy, which was untreated until now as the patient was not showing or talking about any problems. The CP recommended duloxetine … We will check the symptoms and start duloxetine if needed.*	GP 12
	*I prescribed trazodone; the patient didn’t take it out of fear for adverse reactions. The CP proposed mirtazapine or quetiapine instead … The problem is how to convince the patient … I am willing to prescribe any of the options.*	GP 4
	*The patient will take this report to a pulmonologist. It ‘s theophylline in high doses and roflumilast, which I don‘t really know. The medicines were prescribed by pulmonologist, so we will see what he has to say.*	GP 10
	*For this recommendation we decided the patient will consult with the cardiologist.*	GP 8
	*We didn‘t accept this recommendation as her asthma is under control, she practically never uses salbutamol. Therefore, I don‘t feel its necessary. And I prefer to do only one to two changes in therapy at once.*	GP 4
	*I don’t think it’s reasonable to turn whole therapy around to have one medicine less at this high number of medicines. ... we [GP together with the patient] have decided not to switch … The patient is very sensitive about his medicines … he was not very keen on the proposed switch.*	GP 19
	*Sometimes you accept the recommendation, and it confuses the patient. I had cases when it didn‘t work out. He was fine on previous medication when we changed it was not ok.*	GP 20
	*I prescribed the medicine already and wanted to know if it is appropriate. I got confirmation.*	GP 9
	*The report responds directly to the question. First, second and third line of treatment together with dosing are suggested. Even more, the CP proposed combination for other medicines to be joined in one pill.*	GP 9
	*Perfect. Short, to the point, important thing underlined …*	GP 14
**Barriers for the implementation of clinical pharmacists’ recommendations**	*The patient is reluctant to take warfarin from the start, he is actively looking for changes at the skin. And we talked about it several times … but still insists …*	GP 11
	*When it comes to the unfortunate zolpidem … there nothing we can do … This is the therapy from which the patient will not back down …*	GP 13
**Clinical medication review service benefits**		
Assurance of evidence-based pharmacotherapy	*Having a lot of medicines means comorbidity, large number of hurt organ systems and therefore it is always good with such large number of medicines to have professional, evidence-based assessment, and recommendations if we can discontinue some of the medicines.*	GP 21
Benefits for the patients	*It’s a way to get closer to the patients, give them the medicines that suits them and discontinue those, which cause them problems … .*	GP 14
	*It was important to decrease the number of daily doses. The patient was taking medicines four times a day and it was decreased to two.*	GP 7
	*The patient was satisfied and was reassured the medication are not causing any harm and are appropriately chosen.*	GP 1
	*It helps them with adherence as they know how to take the medicines, they discuss and side effects …*	GP 17
	*The CP asks them also about OTCs, food supplements, … We usually don’t ask about it and secondly, we don’t know much about these medicines. The CP counsels them about it – what is reasonable and appropriate to use, what is not, if there are any interaction with regular prescription medicines, …*	GP 7
Benefits for the GPs	*It means the quality of patient care from pharmacological aspect is higher. It gives us confirmation of our work.*	GP 10
	*… for me especially management of patients with multimorbidity. For us, young doctors, these are the biggest challenge to manage … The patients are new to you, with already several prescribed medicines …*	GP 16
	*Even if it’s only medical record review and the CP doesn’t see the patient … I can prescribe the medicines safely.*	GP 14
	*I believe in personal referrals. Sometimes it’s easier just to review records. But when there is conversation with the patient …. more information is gathered.*	GP 3
	*I personally learned a lot and I use the knowledge in everyday practice.*	GP 7
	*It useful because the CP warns us about some things that are maybe not reasonable or recommended, especially in elderly population.*	GP 13
	*Patients are more honest about their medicine taking habits. And about OTCs and food supplements, it‘s new information for us.*	GP 15
	*I heard the last CP’s lecture was a big success. I was unfortunately not there, but colleagues told me it was very good, useful.*	GP 8
	*It happened few times, I detected an issue that the specialist hasn’t and then patients brought them the CMR report, and they implemented recommendations.*	GP 10
**General practitioners’ satisfaction**	*I am very, very satisfied. It‘s a precise, concise review. And it’s simple. It just read it and I don‘t have any reasons not to prescribe as its recommended, because it makes it so simple*	GP 20
**Patients’ satisfaction**	*Patients say great things about it, that she [CP] takes the time for them, she really listens, and they get useful information.*	GP 12

##### Evaluation of the CP recommendations by GPs

In total 112 recommendations were provided, 20% were categorized under the recommendation does not require an intervention or decision of the GP (Table 5). Those were notes or answers to specific GP questions or summary of the information provided to the patients. The overall opinion was that the recommendations are well written, exact, practical, useful and comprehensive., The GP’s expectations were mostly satisfied, sometimes even exceeded. Cases, when expectations were not met, were related to decreasing the number of medicines, which was not possible.

**Table 5:** The acceptance rates and implementation of recommendations by the GPs (*N* = 90)

Acceptance	Implementation	*N*	%
**ACCEPTED**		**55**	**61%**
*Yes*	*implemented*	*22*	*24%*
*Yes*	*to be implemented*	*33*	*37%*
**PARTIALLY OR CONDITIONALLY ACCEPTED**		**23**	**26%**
*Partially*	*implemented*	*3*	*3%*
*Partially*	*to be implemented*	*2*	*2%*
*Partially*	*implemented as needed*	*7*	*8%*
*Conditionally*	*implementation depending on the specialist’s decision*	*7*	*8%*
*Conditionally*	*implementation depending on the patient response*	*4*	*4%*
**NOT ACCEPTED**		**12**	**13%**
*No*	*n/a*	*9*	*10%*
*No, although the recommendation is valid*	*n/a*	*3*	*3%*

Partial or conditional acceptance was related to gradual implementation of recommendation or dependence of the patient reaction to the proposal. When recommendations addressed medicines prescribed by clinical specialist, forwarding the CMR report and leaving the decision to them was the preferred practice. Recommendations were not accepted for the following reasons: stable health status, attachment to certain medication, the benefit of the change is too small, need for additional confirmation, limited number of implemented changes at the same time. In addition, experiences where patients reacted badly to the proposed and implemented changes (issues in medication management or health outcomes) were mentioned. There were also cases, where CMR served as confirmation of already implemented change.

##### Barriers for the implementation of CPs recommendations

Implementation of the CP recommendations is hindered by challenges GPs encounter in patient’s medication treatment: (i) patients have difficulties accepting and adjusting to change in medication, (ii) taking care and prescribing to “inherited patients”, whom they had not yet have the chance to familiarize with (iii) deprescribing or discontinuing hypnotics, sedatives and proton pump inhibitors, (iv) medication adherence and (vi) managing OTC and food supplements with regular prescription medicines.

##### CMR benefits

Assurance of evidence-based pharmacotherapy as the base for effective and safe medication treatment was underlined as the main CMR benefit. After the service patients are empowered in medication taking - personalized medicine plan, reduced number of medicines or doses, decreased concerns and improved adherence. They also receive concrete and practical guidance on medication taking, including OTCs and food supplements.

The main benefit for the GPs was the help with and/or confirmation of the quality of prescribing and patient care, especially for younger, less experienced GPs. CMR is beneficial regardless of the MR type (2b or 3), although it was emphasized that patients’ conversation with the CP adds to the comprehensiveness of the review. Secondly, recommendations regarding dosing regimens, certain patient or medicines groups are concrete, practical, gathered in “one place” and often transferable to other cases. Furthermore, the CMR presents a good and reliable information source regarding medicines and patients. Regular monthly lectures for GPs by the CP, were mentioned as important, educational, and quality part of the service. In addition, benefits are transferable to the clinical specialist, who are sometimes forwarded the report. One participant mentioned, attending the CMR service as an observer was also insightful.

##### GPs’ and patients’ satisfaction

The GPs were very satisfied and consider the CMR service to be very useful. In their experience, patients are also very satisfied, and the service is beneficial for them.

## Discussion

### Main findings

Several positive experiences and high level of accepted or partially accepted CP’s recommendations, suggest CPs’ pharmacotherapy expertise is well accepted and trusted among the participating GPs. The service enhances the quality of patient care and assists GPs’ work, especially through patients’ empowerment in medicines use. In addition, several other benefits for the GPs were recognized (easy, up-to date, reliable information about medicines; transferability of recommendations to similar cases; safe and effective prescribing etc.). Despite all recognized positive outcomes, the level of referrals is still low due to lack of time to recognize patients in need of the service and additional workload to study and implement the recommendations. Suggestions to address these challenges ranged from general improvements of the health system (e.g., more time for the patients, decreased workload, digitalization of medical records) to the improvements at the service level (more specific inclusion criteria, inclusion of nurses in referrals, reminder systems), which are quicker to implement. Additionally, raising awareness among GPs, who do not yet have CMR experiences, is needed.

### Comparison with the existing literature

Findings from this study are consistent with the conclusions of the CMR pilot project evaluation from 2012 [8, 10]. Consistency in findings indicates the CMR service in Slovenia is built on solid foundation, the performance of CMR is comparable and robust to large implementation. Therefore, findings validate the established CMR standard operating procedure and high standards required for service provision in Slovenia (post graduate specialization, additional training, and competencies). Moreover, our findings are coherent with other international studies [6, 16–22, 31–41], specifically studies from UK, the Netherlands and Australia, which also had similar study designs [16, 18–22].

Pharmacotherapy expertise as the main (clinical) pharmacist competency and advantage was discussed in Duncan et al., who underlined MR by GP’s are more time efficient, however pharmacist-led are more thorough due to deeper pharmacotherapy knowledge [18]. GPs in the Netherlands believed their medication knowledge is sufficient, however they also saw the benefit of multidisciplinary teamwork and supported the inclusion of non-dispensing pharmacist in general practice [20]. Moreover, role specification (GP selects the patients and makes clinical decisions, CP provides medication-related recommendations) enables good collaboration between the two complementary healthcare professions as they are guided by the common purpose – providing good and quality patient care [16, 19–21]. Finally, added value of CP pharmacotherapy expertise was noted also for general consultations and queries [16, 19].

Secondly, opinions regarding patient involvement in MR varied. Duncan et al. noted distinction between “simple” review, where patient presence is not necessary and “complex” where patient involvement is merited [18]. GPs in the present study showed similar understanding, however they also noted that patients gain confidence in medicines use after a conversation with the CP and GPs receive new information regarding patient medication related habits, issues, and preferences. Similar conclusions was reached in the systematic review by Willeboordse et al., that showed patient participation results in more identified DRPs, and recommendations related to the identified issues are more likely to be implemented [42]. However, Bajorek et al. reported some patients are reluctant to change, do not perceive the potential benefits of MR and therefore do not wish to attend the MR [16]. High workload and time pressure unable GPs to properly address these issues, which results in MR without patient participation [16, 18, 19]. Similar observations were seen in our study - patient consent for referral was perceived as important, while at the same time lack of time to explain the objectives and benefits of CMR to the patient were also reported as a barrier.

Thirdly, the average acceptance or implementation rate of pharmacist recommendations in the published literature is 50% with large variation between studies [23, 43]. In a systematic review, Kwint. et al., indicated higher implementation rate was related to consistent working relationship between CP and GP with time to establish trust and interprofessional communication [23]. According to this, high acceptance rate (61%) in this study could be partially related to all recommendations being provided by the same CP, who GPs are familiar with and trust. On the other hand, reasons for not accepting certain recommendations are also in line with those reported by Kwint et al. (e.g., patient reluctance to change) [23].

Finally, the relationship between beneficial effect and levels of implementation is sweet-sour. GPs recognize several benefits of CMR, mainly the evidence-based polypharmacotherapy, where medicines use is optimized and tailored for each individual patient [16, 18–21]. These opinions are corroborated with evidence from quantitative studies, showing decreased number of DRPs, controlled blood pressure, cholesterol, etc. [4, 7, 31, 37, 40, 41]. On the other hand, the workload and time pressure unable higher level of referrals to positively impact larger population and consequently unburden the GPs [5, 13, 20]. In their study, Bradely et al. advised that GPs not to expect immediate reductions in workload as the effect of introducing non-dispensing pharmacist is of a long-term nature [22]. The recommendation was provided also considering funding issues, that were reported as barrier in several studies [16, 18–22]. Interestingly, funding issues have not emerged as a theme in the current study.

### Strengths and limitations of the study

The main strength of the study are interviews based on data of the actual patients. Semi structured interviews were chosen as the methodological approach to capture the variety in GPs views and experiences to gain an in depth understanding of the service implementation in practice. Hence, GPs were free to articulate any element related to the CMR service they experience and feel it is important, without being steered into a general understanding, which could happen in focus groups. Furthermore, patient cases mediated the start of the conversation and made it easier for a GP to speak freely and provide concrete experiences. Certain patient problem or characteristic also have recalled similar or contrary experiences from the past providing additional insights. Another strength is the presentation of study findings through Donabedian model, which facilitates the use of findings in future development of the service and practice in general. At the time of writing, we were not aware of similar published studies providing presentation of results through this model.

Several steps in design, conduct and analysis of the study were taken to assure validity of presented results and conclusions according to criteria by Lincoln and Guba [29, 44]. Dependability and credibility were established by combining research and practice experiences from a multidisciplinary research team, which made the study feasible. Potential bias in sampling was limited as the CP enrolled patients prior to the conversation and assessment of their DRPs. Contact with the GPs and interviews were performed only by the researcher to enhance objectivity and additional steps (anonymized and aggregated data) were taken to assure anonymization of participating GPs from colleagues in the research team. The content of interviews and emerging ideas were frequently discussed within the research team during the study period and in post-content analysis phase. Repetitiveness of emerged themes, large number of conducted interviews (*n* = 38) with 24 different GPs indicate data saturation was achieved. Data saturation and similarity of findings from other comparable studies grants confirmability, suggest findings from our study are transferable among different environments and health care systems and can encourage the implementation of CMR in healthcare systems like Slovenian. In addition, several studies evaluating effect of CMR on health outcomes (e.g., decrease in DRPs) also benchmark the qualitative findings from our study.

On the other hand, the CHC Ljubljana employs around 150 GPs and approximately half of them have not referred any patient to the CMR service within the study period. Hence, the results do not encompass the perspective of the GPs, who are reluctant to refer patients and does not provide reasons why they are not using the service. Some indications were provided in the themes covering low level of referrals, however there might be more in-depth reasons behind. Only one CP’s recommendations were part of this study, which can be perceived as a limitation. Different CPs could provide additional variation and potential new insights from the GPs, however we did evaluate the real-world situation, where one CP covered the whole CHC. Furthermore, we cannot fully exclude the potential of observational bias as the CP was aware her work is being evaluated. Other improvements in the study could be made: (i) member checking could be performed as another way of ensuring results credibility, (ii) first coding was performed only by one person (the PhD student), including several researchers would provide additional level of objectivity and understanding.

## Conclusion

GPs experiences with CMR are mainly positive, encouraging, and supportive. CP is recognized as the pharmacotherapy expert and a valued member of the healthcare team with the common goal – providing quality patient care. Currently, number of referrals are low and should be increased with changes and approaches on the CMR service level as well as on the level of healthcare system. Beside evaluating the CMR effect on the health outcomes, future studies should also explore perspective of the GPs, who are reluctant to use the service.

## Supplementary Information

**Additional file 1.** The interview guide.

**Additional file 2.** The COREQ (Consolidated criteria for Reporting Qualitative research) Checklist.

**Additional file 3.** Reasons for exclusion of the patients in the study.

## Data Availability

The datasets generated and/or analysed during the current study are not publicly available as the consent for participation and/or publication provided by patients and GPs does not entail sharing whole interview recordings or transcripts, which represent raw data in this study. If needed, data might be available from the corresponding author on reasonable request.

## Abbreviations

CHC LJ: Community Health Centre Ljubljana
CMR: Clinical medication review
CP: Clinical pharmacist
DRP: Drug related problem
GP: General practitioner
MR: Medication review
OTC: Over the counter medicines
UL FFA: University of Ljubljana, Faculty of Pharmacy
