# Supplementary material for: Pharmacist-led clinical medication review service in primary care: the perspective of general practitioners
Source: BMC Prim Care. 2023 Jan 10;24:6. doi: 10.1186/s12875-022-01963-w (PMC9832745; doi:10.1186/s12875-022-01963-w)
Supplement: Supplementary file 1 — Additional file 1. The interview guide. [file 12875_2022_1963_MOESM1_ESM.docx]

# **Additional file 1**

## The interview guide

5 open ended questions with sub questions:

1. On the referral form, ________ was stated as the reason for referral. Please, provide more details regarding the referral reason?
2. How would you asses the CMR report based on your expectations at the referral of the patient?

- *How does it answer the reason for referral?*
- *How do the proposed recommendations meet your expectations?*
- *How is the CMR report useful for the future treatment of the patient?*

1. Please, outline which CP recommendations will be implemented and what are the reasons for (non)implementation. What will be future steps in patient’s treatment?
   - *How and what changes in treatment will you carry out?*
   - *When will the changes be implemented?*
   - *What other referrals (e.g. lab, clinical specialist…) will be performed?*
   - *Would you recommend a follow up visit at the CP? Please explain.*
2. What benefits of the CMR service and clinical pharmacists’ engagement do you recognize…?

- *… for you as a GP?*
- *…. for the patients?*

1. What improvements would you recommend for the future of the service? Anything else you would like to add to this conversation?
