# Supplementary material for: Pharmacist-led clinical medication review service in primary care: the perspective of general practitioners
Source: BMC Prim Care. 2023 Jan 10;24:6. doi: 10.1186/s12875-022-01963-w (PMC9832745; doi:10.1186/s12875-022-01963-w)
Supplement: Supplementary file 2 — Additional file 2. The COREQ (Consolidated criteria for Reporting Qualitative research) Checklist. [file 12875_2022_1963_MOESM2_ESM.docx]

# **Additional file 2**

## The COREQ (Consolidated criteria for Reporting Qualitative research) Checklist

| **No** | **Item** | **Guide questions/description** |  |
| --- | --- | --- | --- |
| **Domain 1: Research team and reflexivity** | | | |
| **Personal Characteristics** | | | |
| 1. | Interviewer/facilitator | Which author/s conducted the interview or focus group? | See Methods, *Research team* (lines 112-124) |
| 2. | Credentials | What were the researcher's credentials? E.g. PhD, MD | See Methods, *Research team* (lines 112-124) |
| 3. | Occupation | What was their occupation at the time of the study? | See Methods, *Research team* (lines 112-124) |
| 4. | Gender | Was the researcher male or female? | Not specifically reported but can be understood. See Methods, *Research team* (lines 112-124) |
| 5. | Experience and training | What experience or training did the researcher have? | See Methods, *Research team* (lines 112-124) |
| **Relationship with participants** | | | |
| 6. | Relationship established | Was a relationship established prior to study commencement? | See Methods, S*election of participants* (lines 125-135) and Discussion, *Strengths and limitations* (lines 423-448) |
| 7. | Participant knowledge of the interviewer | What did the participants know about the researcher? e.g. personal goals, reasons for doing the research | Not reported |
| 8. | Interviewer characteristics | What characteristics were reported about the interviewer/facilitator? e.g. Bias, assumptions, reasons and interests in the research topic | See Methods, *Research team* (lines 112-124) |
| **Domain 2: study design** | | | |
| **Theoretical framework** | | | |
| 9. | Methodological orientation and Theory | What methodological orientation was stated to underpin the study? e.g. grounded theory, discourse analysis, ethnography, phenomenology, content analysis | See Methods, *Interview guide* (lines 138-145) and *Qualitative analysis* (lines: 155-175) |
| **Participant selection** | | | |
| 10. | Sampling | How were participants selected? e.g. purposive, convenience, consecutive, snowball | See Methods, S*election of participants* (lines 125-135) and *Additional file 3.* |
| 11. | Method of approach | How were participants approached? e.g. face-to-face, telephone, mail, email | See Methods, S*election of participants* (lines 125-135) and Figure 1. |
| 12. | Sample size | How many participants were in the study? | See Results, *Sample size and response rate* (lines 184-193). |
| 13. | Non-participation | How many people refused to participate or dropped out? Reasons? | See Results, *Sample size and response rate* (lines 184-193) and *Additional file 3.* |
| **Setting** | | | |
| 14. | Setting of data collection | Where was the data collected? e.g. home, clinic, workplace | See Methods, *Study design and setting* (lines 105-111) and *Interviews* (lines 146-154) |
| 15. | Presence of non-participants | Was anyone else present besides the participants and researchers? | See Methods, *Research team* (lines 112-124) and *Interviews* (lines 146-154) |
| 16. | Description of sample | What are the important characteristics of the sample? e.g. demographic data, date | See Results, *Characteristic of the participants* (lines 194-197) and *Table 1.* |
| **Data collection** | | | |
| 17. | Interview guide | Were questions, prompts, guides provided by the authors? Was it pilot tested? | See Methods, *Interview guide* (lines 138-145) and *Additional file 1.* |
| 18. | Repeat interviews | Were repeat interviews carried out? If yes, how many? | See Methods, S*election of participants* (lines 125-135), *Interviews* (lines 146-154) and Results, *Sample size and response rate* (lines 184-193) |
| 19. | Audio/visual recording | Did the research use audio or visual recording to collect the data? | See Methods, *The interviews* (lines 146-154) |
| 20. | Field notes | Were field notes made during and/or after the interview or focus group? | See Methods, *The interviews* (lines 146-154) |
| 21. | Duration | What was the duration of the interviews or focus group? | See Results, *Sample and response rate* (lines 184-193) |
| 22. | Data saturation | Was data saturation discussed? | See Discussion, Strengths *and limitations* (lines 423-436) |
| 23. | Transcripts returned | Were transcripts returned to participants for comment and/or correction? | No. See Methods, *Qualitative analysis* (lines: 155-175) and Discussion, *Strengths and limitations* (lines 437-448) |
| **Domain 3: analysis and findings** | | | |
| **Data analysis** | | | |
| 24. | Number of data coders | How many data coders coded the data? | See Methods, *Qualitative analysis* (lines: 155-175) and Discussion, *Strengths and limitations* (lines 437-448) |
| 25. | Description of the coding tree | Did authors provide a description of the coding tree? | See Methods, *Qualitative analysis* (lines: 155-175) and Results, *Results of the qualitative content analysis* (lines 201-205) and Figure 2. |
| 26. | Derivation of themes | Were themes identified in advance or derived from the data? | See Methods, *Qualitative analysis* (lines: 155-175) |
| 27. | Software | What software, if applicable, was used to manage the data? | See Methods, *Qualitative analysis* (lines: 155-175) |
| 28. | Participant checking | Did participants provide feedback on the findings? | No.  See Methods, *Qualitative analysis* (lines: 155-175) and Discussion, *Strengths and limitations* (lines 437-448) |
| **Reporting** | | | |
| 29. | Quotations presented | Were participant quotations presented to illustrate the themes / findings? Was each quotation identified? e.g. participant number | See Results, *Results of the qualitative content analysis* (lines 201- 340) and *Table 2, Table 3 and Table 4* |
| 30. | Data and findings consistent | Was there consistency between the data presented and the findings? | See Results and Discussion section |
| 31. | Clarity of major themes | Were major themes clearly presented in the findings? | See Results and Discussion section |
| 32. | Clarity of minor themes | Is there a description of diverse cases or discussion of minor themes? | See Results and Discussion section |
