# Supplementary material for: Pharmacist-led clinical medication review service in primary care: the perspective of general practitioners
Source: BMC Prim Care. 2023 Jan 10;24:6. doi: 10.1186/s12875-022-01963-w (PMC9832745; doi:10.1186/s12875-022-01963-w)
Supplement: Supplementary file 3 — Additional file 3. Reasons for exclusion of the patients in the study. [file 12875_2022_1963_MOESM3_ESM.docx]

# **Additional file 3**

## Reasons for exclusion of the patients in the study

**Exclusion criteria:**

Patients were excluded if (i) conversation was performed with their carer (ii) attended follow up visit with the CP (iii) their cognitive or physical functions were severely impaired based on CP observation.

**Number of excluded patients with reasons:**

Twenty patients were excluded from the study due to the following reasons:

- 10 patients had decreased cognitive or physical functions (tremor, Parkinson disease, Alzheimer disease)
- 6 patients, where the conversation was performed with relatives or carers (residents of elderly nursing home)
- 3 patients attended the follow up visit
- 1 patient, where the reason is unknown.
